# Non-coding *roX* RNAs Prevent the Binding of the MSL-complex to Heterochromatic Regions

**DOI:** 10.1371/journal.pgen.1004865

**Published:** 2014-12-11

**Authors:** Margarida L. A. Figueiredo, Maria Kim, Philge Philip, Anders Allgardsson, Per Stenberg, Jan Larsson

**Affiliations:** 1 Department of Molecular Biology, Umeå University, Umeå, Sweden; 2 Computational Life Science Cluster (CLiC), Umeå University, Umeå, Sweden; Max Planck Institute of Immunobiology and Epigenetics, Germany

## Abstract

Long non-coding RNAs contribute to dosage compensation in both mammals and *Drosophila* by inducing changes in the chromatin structure of the X-chromosome. In *Drosophila melanogaster*, *roX1* and *roX2* are long non-coding RNAs that together with proteins form the male-specific lethal (MSL) complex, which coats the entire male X-chromosome and mediates dosage compensation by increasing its transcriptional output. Studies on polytene chromosomes have demonstrated that when both *roX1* and *roX2* are absent, the MSL-complex becomes less abundant on the male X-chromosome and is relocated to the chromocenter and the 4^th^ chromosome. Here we address the role of *roX* RNAs in MSL-complex targeting and the evolution of dosage compensation in *Drosophila*. We performed ChIP-seq experiments which showed that MSL-complex recruitment to high affinity sites (HAS) on the X-chromosome is independent of *roX* and that the HAS sequence motif is conserved in *D. simulans*. Additionally, a complete and enzymatically active MSL-complex is recruited to six specific genes on the 4^th^ chromosome. Interestingly, our sequence analysis showed that in the absence of *roX* RNAs, the MSL-complex has an affinity for regions enriched in *Hoppel* transposable elements and repeats in general. We hypothesize that *roX* mutants reveal the ancient targeting of the MSL-complex and propose that the role of *roX* RNAs is to prevent the binding of the MSL-complex to heterochromatin.

## Author Summary

In both fruit flies and humans, males and females have different sets of sex chromosomes. This generates differences in gene dosage that must be compensated for by adjusting the transcriptional output of most genes located on the X-chromosome. The specific recognition and targeting of the X-chromosome is essential for such dosage compensation. In fruit flies, dosage compensation is mediated by the male-specific lethal (MSL) complex, which upregulates gene transcription on the male X-chromosome. The MSL-complex consists of five proteins and two non-coding RNAs, *roX1* and *roX2*. While non-coding RNAs are known to be critical for dosage compensation in both flies and mammals, their precise functions remain elusive. Here we present a study on the targeting and function of the MSL-complex in the absence of *roX* RNAs. The results obtained suggest that the dosage compensating MSL-complex has an intrinsic tendency to target repeat-rich regions and that the function of *roX* RNAs is to prevent its binding to such targets. Our findings reveal an ancient targeting and regulatory function of the MSL-complex that has been adapted for use in dosage compensation and modified by the rapidly evolving noncoding *roX* RNAs.

## Introduction

In many animal species with distinct sexes, sex-chromosomes contribute to genetic sex determination. In species with male heterogamety such as humans and fruit flies, the male sex-chromosome pair consists of two morphologically and genetically different chromosomes (one X and one Y) whereas females are homogametic, having two X chromosomes. The heteromorphic sex-chromosomes are believed to have evolved from a pair of autosomes in which a male-determining locus was acquired on one homolog to form a proto-Y chromosome that subsequently underwent a series of mutation and selection events that conferred male advantage and suppressed recombination with the proto-X, eventually leading to the degeneration of the Y-chromosome. Gene expression imbalances arise because X-chromosomal genes in male genomes are only present in one copy whereas autosomal genes and X-chromosomal genes in females are present in two copies. Dosage compensation mechanisms evolved in order to balance the relative expression levels of X-chromosomal genes between the sexes and in relation to autosomal genes [1]–[3].

Dosage compensation in *D. melanogaster* involves a combination of general buffering effects that act on all monosomic regions [4]–[6] and the specific targeting and stimulation of the male X-chromosome by the male-specific lethal (MSL) complex. Together, these processes increase X-chromosomal gene expression by approximately a factor of two [1], [7]. The MSL-complex consists of five proteins (MSL1, MSL2, MSL3, MLE, and MOF) and two redundant long non-coding RNAs (*roX1* and *roX2*) [7]–[9]. It is believed that the hypertranscription of the male X-chromosome is partly due to the enrichment of histone 4 lysine 16 acetylation (H4K16ac). This acetylation is catalyzed by the acetyltransferase MOF and opens the chromatin's structure [10], [11]. The complete MSL-complex only forms in males due to the male-specific expression of MSL2 and the *roX* RNAs [12]–[16]. Notably, even though most genes on the X-chromosome appears dosage compensated in the 2-fold range [17], [18] the MSL-complex only contributes to part of this increase [6], [19], [20]. In addition, many genes are compensated without any significant recruitment of the MSL-complex [17]. An alternative model for the role of the MSL-complex in dosage compensation has been proposed by Birchler and colleagues [21]–[23]. According to their inverse dosage effect model the compensation in males is caused by the stoichiometric change of regulator(s) on the X-chromosome relative to the remainder of the genome. The main role of the MSL-complex is to sequester MOF from the autosomes to avoid autosome up-regulation and to limit the activation potential of MOF when targeted as part of the MSL-complex [21]–[24].

It is still not clear when, where and how the MSL-complex is assembled or which features of the X-chromosome allow its recognition. Several lines of evidence indicate that MSL1 and MSL2 are the core components of the MSL-complex. Notably, the absence of either one abolishes the binding of the remaining components of the complex to the X-chromosome [25]. The RING domain of MSL2 allows it to interact with MSL1, and the cystein-rich (CXC) domain of MSL2 allows the MSL1-MSL2 complex to recognize and bind DNA [26], [27]. The incorporation of *roX* RNAs into the MSL-complex is hypothesized to occur co-transcriptionally [28] and depends on their interaction with MSL2 and the RNA helicase MLE, which binds to stem-loop structures on *roX* RNAs in an ATP-dependent manner [29]–[32].

The *roX1* and *roX2* gene loci have been identified as two of the strongest high affinity sites (HAS) for MSL-complex targeting, out of the roughly 250 HAS on the X-chromosome [33], [34]. HAS are defined as sites targeted by MSL1 and MSL2 in the absence of *msl3*, *mle* or *mof* [25], [34], [35] and sites that are sufficient to recruit MSL even when inserted on an autosome [36]. HAS are enriched in a conserved consensus GA-rich DNA sequence motif [34], [37], [38]. The prevailing model is that the MSL-complex initially binds at the HAS and that its presence at these sites facilitates the more transient binding of additional MSL-complexes to neighboring active genes [8], [9], [39]. The transcriptional statuses of X-chromosomal genes influence the distribution of MSL binding because the complex is biased to exons and the 3′ ends of actively expressed genes; its binding correlates with enrichment in histone 3 lysine 36 trimethylation (H3K36me3) [34], [37], [40]–[43]. Other features such as the local chromatin context, H3 depletion, MSL-complex concentration, levels of affinity and sequence composition also contribute to the recognition and spreading of the MSL-complex over the male X-chromosome [35], [38], [44]–[46].

The roles of the two redundant long non-coding RNAs, *roX1* and *roX2*, in the targeting of the entire male X-chromosome by the MSL-complex are not fully understood at present. Studies on polytene chromosomes have shown that in the absence of both *roX1* and *roX2*, MSL2 and H4K16ac become less abundant on the male X-chromosome, with the MSL-complexes being relocated to the chromocenter, the 4^th^ chromosome and a few other autosomal sites [20], [47], [48]. In this work, we analyzed MSL-complex targeting in *roX1 roX2* mutants in order to unravel the specific roles of *roX* RNAs in MSL targeting and to learn more about the evolution of chromosome-specific targeting and dosage compensation. We performed ChIP-seq and cytological analyses of MSL proteins in *roX1 roX2* double mutants, and analyzed their genome-wide binding profiles. It was found that in the absence of *roX* RNAs, the MSL-complex binds to the previously identified HAS on the X chromosome, the pericentromeric regions of all chromosomes, and specifically to six genes on the 4^th^ chromosome. Analysis of the autosomal sequences bound by MSL in *roX* mutants showed that MSL has an affinity for regions enriched in *Hoppel* transposable elements, *NTS* (non-transcribed spacers) and repeats. Our results suggest that one role of the *roX* RNAs is preventing the MSL-complex from binding to heterochromatic repeats, suggesting that targeting heterochromatin is an intrinsic and ancient property of the MSL-complex.

## Results

### The MSL-complex is relocalized to heterochromatin in *roX* mutants

**Figure 1:**
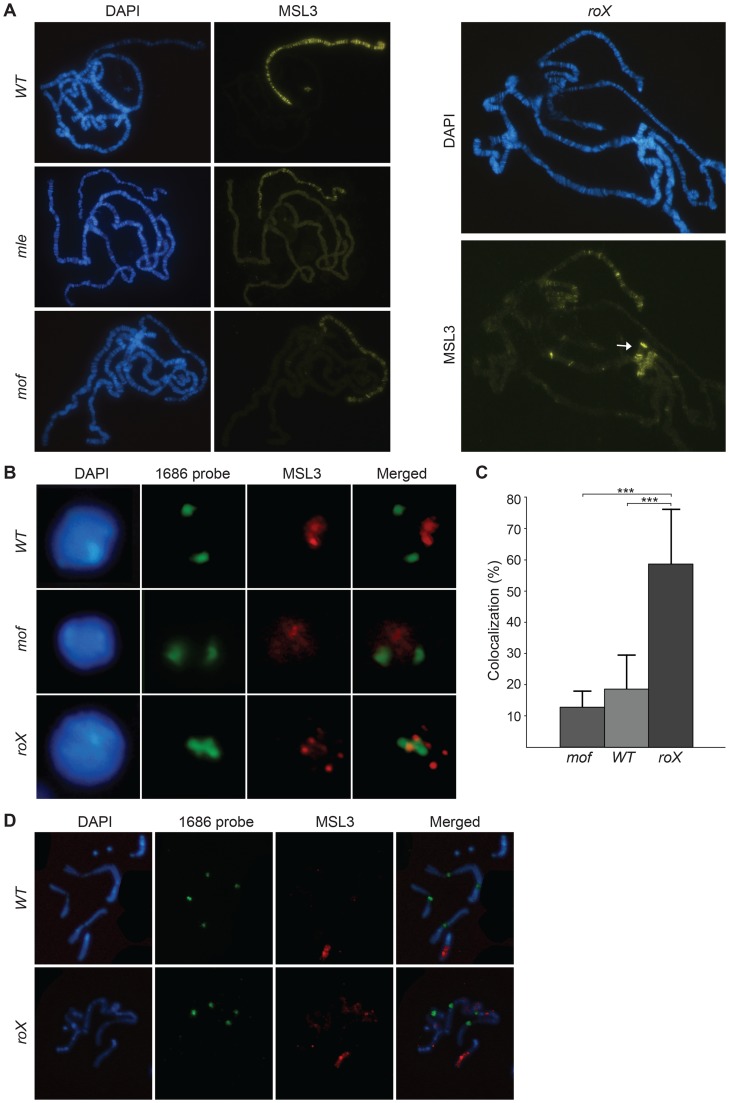
The MSL-complex is redistributed in *roX* mutants. (A) MSL3 immunostaining on polytene chromosomes from wild type, *mle*, *mof* and *roX* mutant males. Note that MSL3 only targets a subset of sites on the X-chromosome, the 4^th^ chromosome and the chromocenter (indicated by the arrow) in *roX* mutants. (B) DNA-FISH with a probe against the 1.686 g/cm^3^ satellite repeat from pericentromeric regions of chromosomes 2 and 3 (1686 probe) combined with MSL3 immunostaining, on interphase nuclei of brain cells from third instar male larvae wild type, *mof* and *roX* mutants. (C) Percentage colocalization of 1686 probe signal and MSL3 staining from 8 replicates each of the genotypes: wild type, *mof* and *roX* (30–50 nuclei scored per replicate). The bars indicate the mean colocalization and the whiskers indicate standard deviations. Significant differences are indicated by *** (Independent two-sample t-test, *p*<0.001). (D) DNA-FISH with the 1686 probe combined with MSL3 immunostaining on metaphase nuclei of brain cells from third instar larvae wild type and *roX* mutant males. Note that on metaphase chromosomes MSL3 colocalization with centromeres is not detected in *roX* mutants.

To test the role of *roX* RNAs in MSL targeting, we performed immunostaining experiments on polytene chromosomes of *roX1 roX2* double mutants (hereafter called *roX* mutants). In the absence of *roX* RNAs, the extent of MSL-complex targeting to the X-chromosome was dramatically reduced and the complex was relocalized to the chromocenter and to three distinct regions on the 4^th^ chromosome (Fig. 1A). The disruption of MSL targeting seen in *roX* mutants is clearly different from the disturbance that occurs when the protein components of the complex are removed: in *msl1* or *msl2* mutants, no MSL-complexes are formed on the X-chromosome at all [25]. Conversely, as shown in Fig. 1A, in *mle* or *mof* mutants, the MSL-complex is exclusively targeted to a limited number of bands on the X-chromosome. This shows that the *roX* RNAs and the protein components of the complex have different functional roles in MSL chromatin targeting.

To exclude the possibility that the binding of the MSL-complex to pericentromeric heterochromatin in *roX* mutants is unique to polytene chromosomes, we analyzed MSL binding in relation to the pericentromeric repeat 1.686 (which is known to be enriched in the pericentromeric regions of chromosomes 2 and 3 [49]) in interphase nuclei from brain cells of wild type samples and *roX* mutants. In the wild type and *mof* mutants, the MSL3-bound X-chromosome occupies a part of the nucleus that is clearly separated from the pericentromeric regions (Fig. 1B and 1C). In *roX* mutants, the normal binding of MSL3 is altered and the complex is observed in spots that colocalize with the centromeric repeats. This colocalization is three times more frequent in *roX* mutants than in wild type or *mof* mutants (Fig. 1C).

We therefore conclude that the relocalization of MSL in the absence of *roX* RNAs observed in salivary gland nuclei also occurs in diploid interphase nuclei. Interestingly, MSL binding in metaphase chromosomes of wild type and *roX* mutants is similar and is restricted to the euchromatic part of the X-chromosome (Fig. 1D). It remains to be determined why MSL doesn't target centromeres in the highly compacted mitotic chromatin in *roX* mutants.

### MSL recruitment to High Affinity Sites (HAS) is independent of *roX*

**Figure 2:**
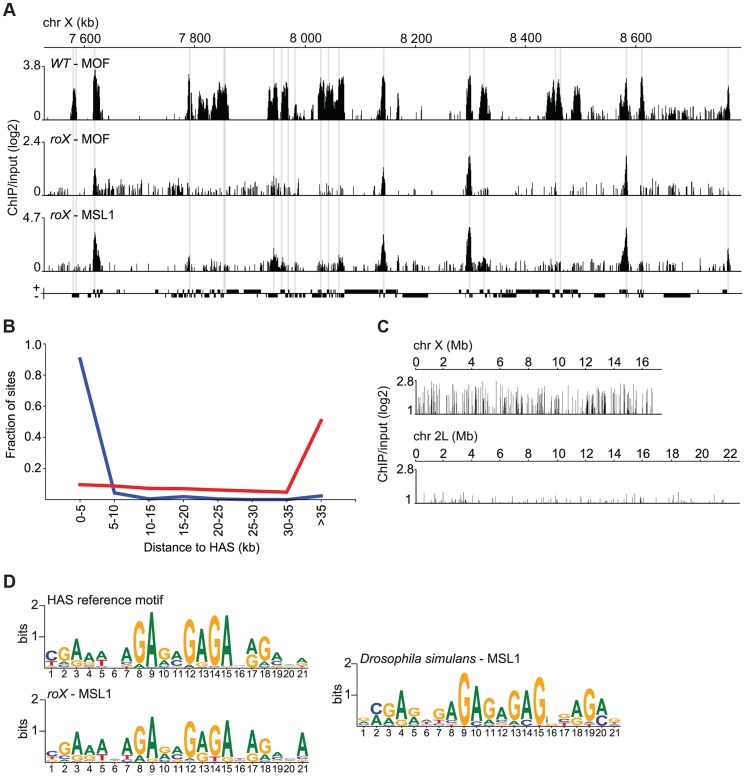
MSL recruitment to the conserved HAS is independent of *roX.* (A) MOF and MSL1 ChIP-seq enrichment profiles, with a 500 bp smoothing, for a representative region of the X-chromosome in salivary gland tissue from wild type and *roX* mutant males. Numbers along the x-axis denote positions along the chromosome in kb. The y-axis shows the ChIP enrichment over input as log2 ratios. Genes expressed from left to right and vice versa are shown above and below the horizontal lines, respectively. The HAS locations, previously defined by [34], [37], [38], are indicated by grey boxes. (B) Fraction of sites on the X-chromosome from *roX* mutants that are bound by MSL1, compared to random sites along the X-chromosome (red), sorted by distance to HAS. (C) MSL1 ChIP-seq enrichment profile, with a 500 bp smoothing, for the entirety of chromosomes X and 2L in salivary glands from *D. simulans* wild type males. Numbers along the x-axis denote chromosomal positions in Mb. The y-axis shows the ChIP enrichment over input as log2 ratios. (D) Sequence motifs enriched in MSL-bound regions of the X-chromosome in *D. melanogaster* wild type [37], *roX* mutants and *D. simulans* wild type.

To better understand how the genome-wide targeting of MSL depends on *roX* RNAs, we performed MSL1, MSL2 and MOF ChIP-seq experiments on salivary glands from wild type individuals and *roX* mutants. These experiments confirmed the results of the immunostaining studies, showing that there is a pronounced decrease in MSL binding along the X-chromosome in *roX* mutants although binding persists at specific locations. Visual inspection demonstrates that the MSL enrichment peaks along the X-chromosome in *roX* mutants coincide with the previously defined HAS (Fig. 2A) [34], [37], [38]. Notably, although all previously defined HAS are not recognized by MSL enrichment in *roX* mutants, all enrichment peaks coincide with HAS. To verify that the *roX* RNA-independent MSL enrichment peaks on the X-chromosome correspond to the previously mapped HAS, we calculated the shortest distance between the coordinates of the HAS and those of the MSL1 binding sites on the X-chromosome in *roX* mutants identified in our ChIP-seq experiments. The fractions of sites bound by MSL1 in *roX* mutants were plotted against distance to nearest HAS, and the distances between the coordinates of HAS and random positions on the X-chromosome were used as controls. As seen in Fig. 2B, the largest fraction of the MSL binding sites in *roX* mutants overlap with HAS. This is in clear contrast to the control, in which the largest fraction of random X-chromosome sites are>35 kb away from HAS. Taken together this means that although all of the 263 previously defined HAS are not bound by MSL in *roX* mutants, in principle all of our 208 defined MSL binding sites in *roX* mutants target HAS. These results demonstrate that MSL binding to HAS on the X-chromosome occurs independently of *roX* RNAs.

It has been shown that *roX* RNAs evolve rapidly, only sharing about 90% and 80% sequence homology in such closely related species as *D. simulans* and *D. yakuba*, respectively [30]. We therefore sought to determine whether HAS, previously shown to be enriched in a GA-rich motif [37], [38], are under high evolutionary pressure. To facilitate comparison with other *Drosophila* species, we generated ChIP-seq data for MSL1 binding in wild type *Drosophila simulans* and performed a motif analysis in the MSL1-bound regions on the X-chromosome of this species (Fig. 2C). We found highly similar GA-rich motifs to be enriched within MSL targets on the X-chromosome in *roX* mutants as well as on the X-chromosome of wild type *D. simulans* (Fig. 2D).

These results show that the *roX* RNAs are not involved in MSL targeting to HAS and that the HAS motif is evolutionarily conserved.

### A complete and enzymatically active MSL-complex is assembled in *roX* mutants

**Figure 3:**
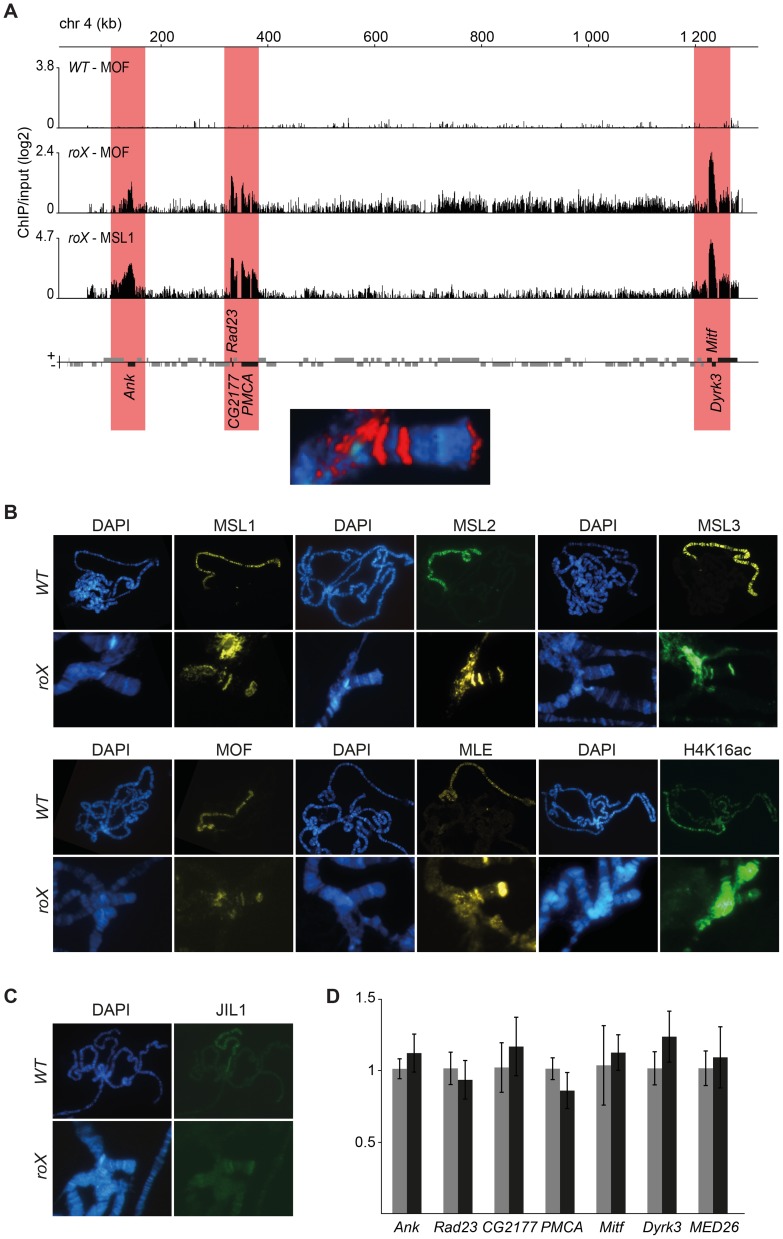
A complete and enzymatically active MSL-complex is assembled in the absence of *roX.* (A) MOF and MSL1 ChIP-seq enrichment profiles, with a 500 bp smoothing, for the entire 4^th^ chromosome in salivary gland tissue from *wild type* and *roX* mutant males. Numbers along the x-axis denote chromosomal positions along the chromosome in kb. The y-axis shows the ChIP enrichment over input as log2 ratios. Genes expressed from left to right and vice versa are shown above and below the horizontal lines, respectively. Note that the genes targeted in *roX* mutants (indicated by red boxes) correspond to the three bands seen in polytene chromosome staining (below). (B) MSL1, MSL2, MSL3, MLE, MOF and H4K16ac immunostaining on polytene chromosomes from wild type males, with X-chromosome targeting, and from *roX* mutant males, showing the 4^th^ chromosome and chromocenter targeting. (C) JIL1 immunostaining on polytene chromosomes from wild type and *roX* mutants males, shows targeting to the chromocenter and to the same chromosome 4 bands as the MSL-complex. (D) Mean levels of mRNA from the six genes targeted by MSL in *roX* mutants and from a control gene on the 4^th^ chromosome that is not bound by MSL (*MED26*), determined by rt-qPCR (black). The corresponding mean expression of the same genes in wild type is shown in grey. The mRNA levels measured by qPCR were normalized against *RpL32* mRNA in each replicate. Error bars represent the standard deviation of three biological replicates.

The binding of MSL to the 4^th^ chromosome in the absence of *roX* RNAs is intriguing because there are several lines of evidence suggesting an evolutionary relationship between the 4^th^ chromosome and the X-chromosome [1], [50]–[52]. Our ChIP-seq profiles show that the MSL-complex binds specifically to six genes on the 4^th^ chromosome in *roX* mutants: *Ankyrin*, *Rad23*, *CG2177*, *PMCA*, *Mitf* and *Dyrk3*. The locations of these genes correspond to those of the MSL-stained bands seen on polytene chromosomes (Fig. 3A). One important question when considering the binding of MSL outside the X-chromosome is whether a complete and functional MSL-complex is formed at these locations. Our immunostaining experiments in *roX* mutants showed that all of the complex's protein components (MSL1, MSL2, MSL3, MLE and MOF) colocalize perfectly at the chromocenter and at the three bands on the 4^th^ chromosome (Fig. 3B). In addition H4K16ac is also enriched at these three bands in *roX* mutants, which indicates that the MSL-complex is complete and active (Fig. 3B and S1 Figure). Note that H4K16ac on the 4^th^ chromosome shows a broader enrichment pattern compared to the MSL proteins in similarity to what previously have been observed for H4K16ac in relation to MSL on the male X-chromosome in wild type [10]. Next we tested the H3S10 kinase JIL1, previously shown to be enriched on the male X-chromosome and dependent on a functional MSL-complex for its targeting [53]–[55]. JIL1 has previously been shown to co-immunoprecipitate with the MSL-complex under low stringency conditions or after formaldehyde cross-linking [54], [56]. Interestingly, like the MSL-complex, JIL1 is also relocalized to the chromocenter and the three regions on the 4^th^ in the absence of *roX* RNAs (Fig. 3C).

Since H4K16ac overlaps with all the other proteins from the MSL-complex in *roX* mutants we asked if the six identified genes on the 4^th^ bound by MSL in *roX* mutants have higher transcriptional output than in wild type. The relative expression of the six 4^th^ chromosome MSL-bound genes was not found to differ significantly between wild type and *roX* mutants (Fig. 3D).

One tempting hypothesis based on the targeting of the 4^th^ chromosome is that the MSL-complex in *D. melanogaster* still has an affinity for ancestral X-chromosomal sequences, now present on the 4^th^. We performed BLAST searches for the sequences of the six 4^th^ chromosome genes targeted by MSL in *D. melanogaster roX* mutants in the distantly related species *D. virilis* and *D. willistoni* and found that in both species, these six genes are assigned to the sequence scaffold on which all of the other 4^th^ chromosome-linked genes are located rather than to the X chromosome. In the even more distantly related species *D. busckii*, the whole correspondent to the 4^th^ chromosome of *D. melanogaster* is fused to the X-chromosome [57], [58]. Interestingly, it has recently been shown [50] that in *D. busckii* the sequences corresponding to the *D. melanogaster* 4^th^ chromosome are present in more copies in females than males. However, the female-to-male ratio is less than 2 meaning that the corresponding homologs on the Y-chromosome are not fully degenerated or that some but not all genes on the corresponding 4^th^ chromosome have degenerated homologs on the *D. busckii* Y-chromosome. We hypothesized that the six genes targeted by MSL in *roX* mutants actually skew the ratio and therefore calculated the female-to-male ratio of these *D. busckii* orthologs relative to the other chromosome 4 genes. By using previously reported data [50] we found that there is no significant difference between the 6 genes and the other 4^th^ chromosome genes (S2 Figure). We conclude that a complete and active MSL-complex binds with high specificity to six genes on the 4^th^ chromosome, although the reason for this specificity remains elusive.

### In the absence of *roX,* MSL has affinity for *Hoppel* transposable elements

**Figure 4:**
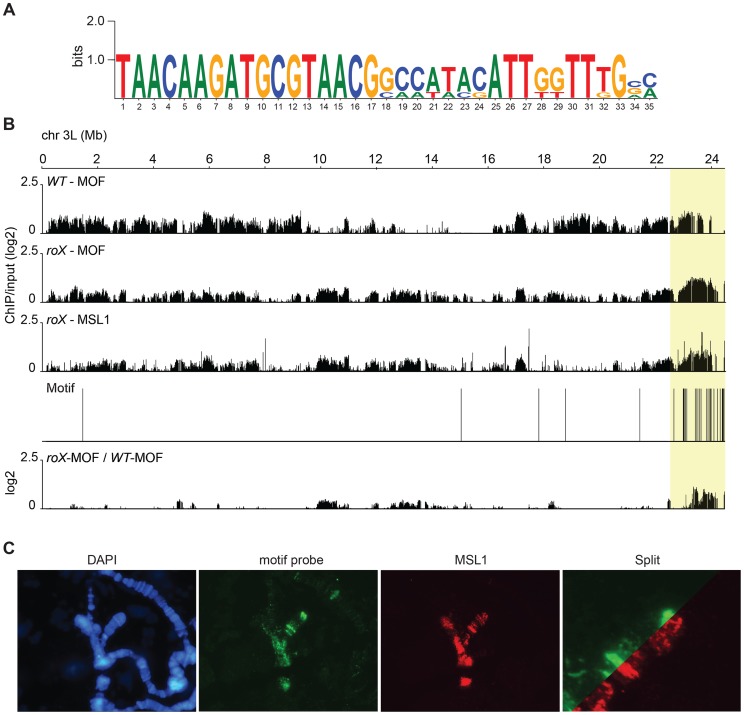
In *roX* mutants *Hoppel* is enriched in MSL-complex. (A) Sequence motif enriched in MSL-bound regions on heterochromatin of *roX* mutants. (B) MOF and MSL1 ChIP-seq enrichment profiles, with a 2000 bp smoothing, for the representative chromosome arm 3L from wild type and *roX* mutant males. Numbers along the x-axis denote chromosomal positions along the chromosome in Mb. The y-axis shows the ChIP enrichment over input as log2 ratios. The mapping of the motif along 3L is indicated. Below the motif track the ratio *roX*-MOF/WT-MOF is plotted. Note that in *roX* mutants MSL is enriched in the pericentromeric region (indicated by a yellow box). (C) DNA-FISH with a 33 nucleotide long probe against the heterochromatic motif shown in A combined with MSL1 immunostaining, on polytene chromosomes of *roX* mutant males. Note the overall colocalization between the MSL1 bound regions and the motif hybridisation.

The pericentromeric regions and the 4^th^ chromosome are both heterochromatic regions of the *D. melanogaster* genome that are targeted by MSL in *roX* mutants and are enriched in satellite repeat sequences, transposable elements, and the heterochromatic proteins HP1a and HP2, among others. Our ChIP-seq results show that in the absence of *roX* RNAs, the MSL-complex targets the pericentromeric regions of all chromosomes and that its abundance increases gradually on moving towards the centromere. This tendency is illustrated for chromosome 3L in Fig. 4B. One possible mechanism underlying this binding is that MSL recognizes a specific recruitment element but that its binding to this element is blocked by the presence of *roX* RNAs. Another possibility is that the MSL-complex has an intrinsic affinity for heterochromatic sequences or repeats in general. Using the MEME software, we analyzed the identified MSL1-bound heterochromatic sequences of each chromosome (2LHet, 2RHet, 3LHet, 3RHet, 4Het, XHet). A specific motif corresponding to repeats in the *Hoppel* (*1360*) transposable element was found to be significantly enriched (Fig. 4A and B). *In situ* DNA hybridization experiments using the identified motif as a probe in conjunction with MSL staining revealed a high degree of colocalization between the MSL-complex and the motif in both the pericentromeric heterochromatin and the three specific bands on the 4^th^ chromosome (Fig. 4C). To determine whether this motif can act as an MSL-recruitment element, we generated a construct containing three tandemly repeated copies of a 108-nucleotide *Hoppel* element featuring the motif in question. This repeat segment was placed upstream of a cDNA copy of *ankyrin* (a gene on the 4^th^ targeted by MSL in *roX* mutants) under an endogenous promoter. The construct was inserted into the 3L:65B2 PhiC landing platform and tested for MSL binding in a *roX* mutant background. The transgene was visualized using mini-*white* DNA-FISH and MSL-complex was not detected on the target (S3 Figure). These results suggest that the repeat motif from the *Hoppel* transposable element, which is enriched at MSL-targeted regions (4^th^ and pericentromeric) in *roX* mutants, is not by itself sufficient to recruit the MSL-complex. However, we cannot exclude the possibility that recruitment might be achieved with a greater number of motif copies.

**Figure 5:**
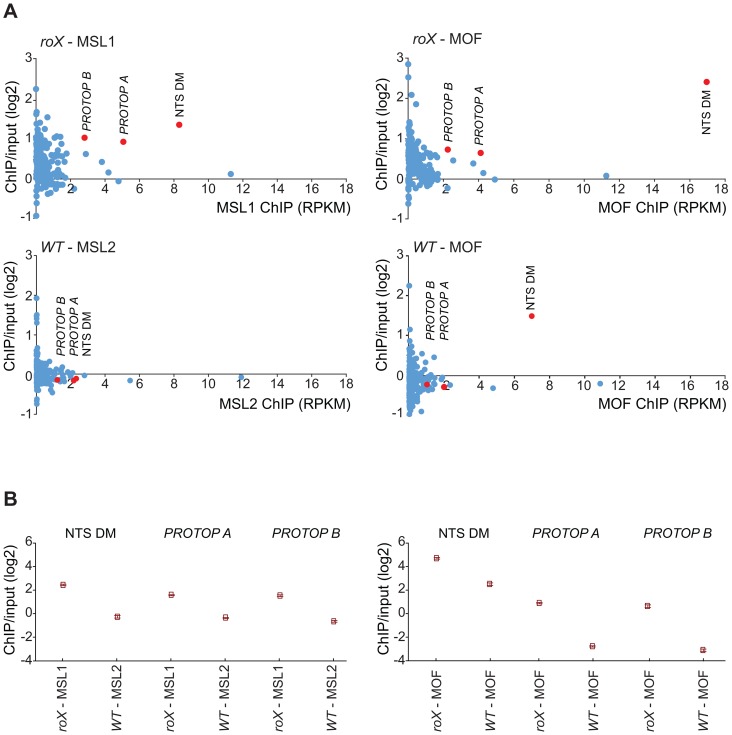
MSL-complex targets *PROTOP* and *NTS* in *roX* mutants. (A) Reads from the ChIP samples mapped to repeat classes from Repbase Update are shown on the x-axis as RPKM values (*R*eads *P*er *K*ilobase per *M*illion mapped reads) and related to ChIP/input enrichment ratios, which are shown on the y-axis. The left part shows MSL1 ChIP in *roX* mutants compared to MSL2 in wild type and the right part shows MOF ChIP in *roX* mutants and wild type. Note, that *NTS* is enriched also in MOF wild type. (B) Enrichment ratio (IP/input) of the overrepresented repeats from A (*NTS*, *PROTOP_A*, *PROTOP_B*) in *roX* mutants compared to the wild type. Rectangles represent the mean values of the IP/input enrichment ratios of all mapped and unmapped reads matching to each repeat type, and error bars indicate 95% confidence intervals.

DNA sequences from centromeres, telomeres, the Y-chromosome and other heterochromatic regions are not assembled to any region of the *D. melanogaster* genome due to their highly repeated nature, and the mapping of sequences recovered in the ChIP-seq normally discards the large number of repeated sequences in the genome. We suspected that the non-mapped reads recovered by ChIP-seq might hold information about other transposable elements targeted by MSL in *roX* mutants in the above-mentioned heterochromatic regions. To test this hypothesis, we aligned all of the ChIP-seq reads to the repeat class sequences from the Repbase Update database and calculated RPKM values for each repeat class. Using this approach we found that in *roX* mutants there were strong enrichments of three repeat classes: *PROTOP_B*, *PROTOP_A* and *NTS* (Non-transcribed Spacer) (Fig. 5). Interestingly, the *PROTOP* is a family of autonomous DNA transposons that have been suggested to be ancient ancestors of the P-element and *Hoppel* element transposon families [59] and *PROTOP_A* and *PROTOP_B* are listed as synonyms of *Hoppel* [60]. Our results confirm that MSL has an affinity for regions enriched in repeats from *Hoppel* and *PROTOP* transposable elements and for *NTS*. All of these are highly repeated elements that are present in heterochromatic regions of the genome.

### The MSL-complex has an affinity for repeat enriched regions

**Figure 6:**
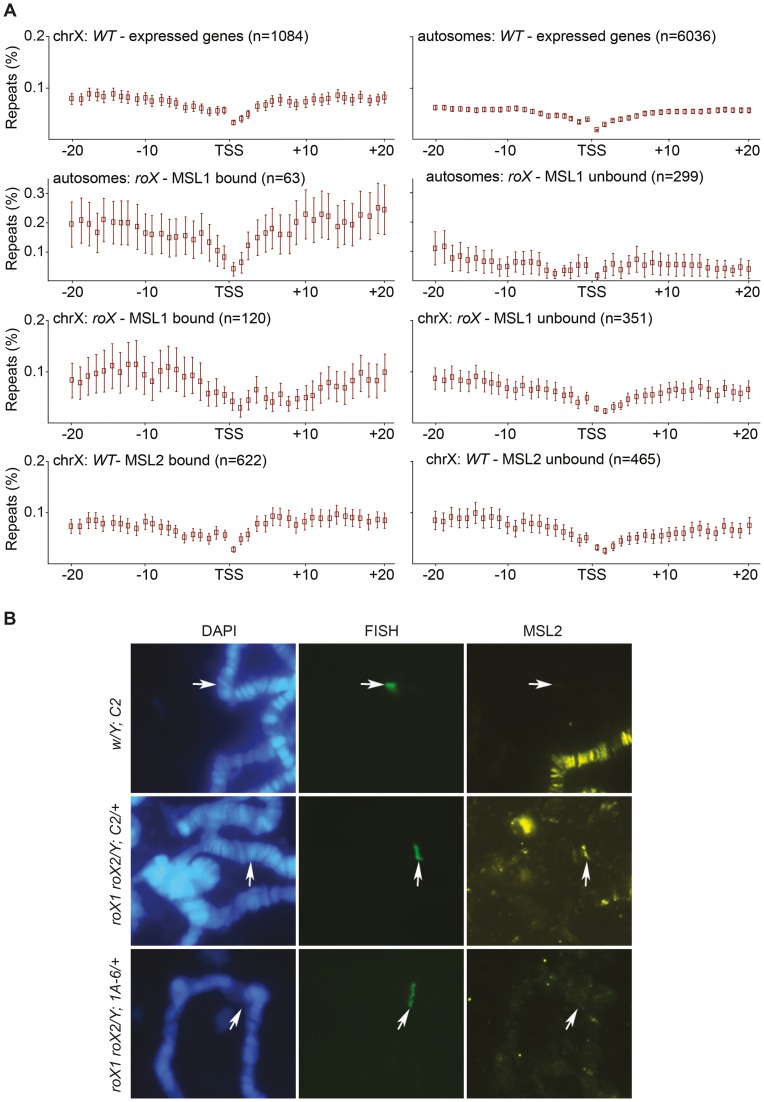
MSL-complex has affinity to repeats. (A) Percentage of repeat masked sequences (from UCSC), in 20 bins of 1 kb distance from the transcription start site (TSS) of X-chromosome expressed genes, autosomal expressed genes, MSL1-bound/unbound genes in *roX* mutants, on the X-chromosome and on autosomes. Rectangles represent the mean values of repeats for all expressed genes in each distance bin, and error bars indicate 95% confidence interval. (B) DNA-FISH with a probe against the mini-*white* gene combined with MSL2 immunostaining, on polytene chromosomes of wild type and *roX* mutant males. *C2* is a cluster of seven tandemly repeated copies of *P[lacW]* transgene, the 1A-6 cluster consists of two copies. Note that the transgene cluster consisting of seven copies recruits MSL2 in a *roX* mutant background. The site of the construct insertion is indicated by arrows.

In addition to the *Hoppel* transposable element repeats, the analysis of the mapped and unmapped sequences bound by MSL in *roX* mutants recovered *NTS* sequences, which occur between ribosomal DNA genes which are organized in tandem repeats. Because MSL targets some autosomal sites across the genome in the absence of *roX*, we wondered whether these sites were also enriched in repeats and whether repeats in general were enough to recruit MSL. To test this hypothesis, we analyzed the enrichment of repeat masked sequences around MSL targeted regions. Since the MSL-complex mainly targets expressed genes we calculated the enrichment of repeats surrounding the TSS (transcription start site) of genes that are expressed in salivary glands (the tissue of our binding data) and are located in either MSL-bound or MSL-unbound regions of the genome. We examined both the X-chromosome and the autosomes (excluding chromosome 4 and the mapped pericentromeric regions) in this analysis, taking into account the MSL-bound regions in wild type and *roX* mutants that were identified based on our ChIP-seq data. The density of satellite repeats on the X-chromosome is reportedly greater than on the 2^nd^ and 3^rd^ chromosomes [61]–[63]. Our results are consistent with this finding and show that the regions surrounding expressed genes on the X-chromosome have a somewhat higher repeat content than those surrounding autosomal expressed genes (Fig. 6A). Strikingly, autosomal expressed genes bound by MSL in *roX* mutants are enriched in surrounding repeats whereas the regions surrounding unbound autosomal expressed genes have a low repeat content. The repeat content of regions surrounding X-chromosomal genes bound by MSL was also higher than that of unbound regions, but the difference was less pronounced than for autosomal genes. Our results show that MSL targeting of autosomal sites in *roX* mutants correlates with high repeat content.

We therefore sought to determine whether any repeat sequence would recruit MSL in the absence of *roX*. Two repeat types were tested. First we analyzed clusters of tandemly repeated *P[lacW]* transgenes on the 2^nd^ chromosome in *roX* mutants [64]. The *P[lacW]* transgene contains sequences of the P-element flanking the *E. coli lacZ* gene and the mini*-white* gene. In the absence of *roX,* MSL2 did indeed bind to a cluster of 7 tandemly repeated copies of *P[lacW]* (*C-2*, *BX-2*, *T-1*) but not to a cluster with only 2 copies (*1A-6*) (Fig. 6B). Although mini*-white* originates from an X-linked gene, it does not contain HAS and is not an ectopic MSL target in the wild type. These results show that genes in tandem repeats are enough to recruit the MSL-complex when *roX* RNAs are absent. The second repeat cluster tested in a *roX* mutant background was the 256 *lac* repeats of *E. coli* upstream of a *white* reporter gene [65]. In this system, the DNA binding domain of the *lac* repressor (*lacI* BD) fused to HP1a is tethered to a reporter transgene that contains repetitive binding sites for the *lacI* BD (*lacO* repeats). The transgene was targeted by HP1a in about 50% of nuclei but never by MSL3 (S4 Figure).

Overall, these results suggest that in absence of the *roX* RNAs, MSL has an intrinsic general affinity for repeated sequences. However, the influence of the repeat length and number of copies as well as the sequence specificity of the complex all remain to be elucidated.

## Discussion

The correct functioning of dosage compensation mechanisms in both mammals and flies is dependent on the presence of non-coding RNAs [66]. Although the *roX* genes are known to be important for MSL-complex targeting, their function in this process and the targets of the complex in the absence of *roX* RNAs were previously unknown. In this work we used immunostaining and ChIP-seq techniques to map the targets of the MSL-complex in the absence of *roX*. Based on the results of these experiments, we present a model that describes the role of the non-coding *roX* RNAs in targeting the MSL-complex to the male X-chromosome.

### *roX* RNAs prevent the targeting of heterochromatin by the MSL-complex

We and others have previously shown that in the absence of *roX* the MSL-complex is redistributed to targets corresponding to pericentric heterochromatin and the 4^th^ chromosome, or “green chromatin” according to recent chromatin structure-based definitions [20], [47], [48], [67]. Since large parts of these “heterochromatic” regions targeted by MSL in *roX* mutants are under-replicated in polytene chromosomes it was important to determine whether this redistribution also occurs in diploid cells that have very different ratios of the relevant DNA motifs. Notably, although the reduction in MSL-complex abundance on the X-chromosome is much more dramatic in *roX* mutants than in *mle* or *mof* mutants and the MSL-complex is relocalized to heterochromatic regions in *roX* mutants, escaping males are recovered in *roX* mutants in contrast to the complete male lethality observed in *mle*, *msl3* or *mof* mutants [47], [68], [69]. The results of our studies on interphase nuclei from brain tissue showing colocalization between MSL3 and centromeric regions further support the interpretation that in absence of *roX*, the MSL-complex targets heterochromatin.

It has previously been shown that the fraction of escaper males in *roX* mutants is significantly higher in *roX1 roX2/0* males, *i.e.* males lacking a Y-chromosome, than in *roX1 roX2/Y* males. Importantly, the Y-chromosome is predicted to be 40 Mb in length and thus accounts for>10% of all genomic DNA in male cells [70]. It seems likely that the reduced abundance of heterochromatic target DNA and/or the greater compaction of the remaining heterochromatin caused by the loss of the Y-chromosome increases the X-chromosomal targeting of MSL in *roX1 roX2/0* males, explaining their increased survival. It is tempting to speculate that in *roX* mutant interphase nuclei, the centromeric regions of autosomes have a tendency to colocalize with the X-chromosome within the nucleus, in a region where the local concentration of the MSL-complex is expected to be high. In fact, previous studies have shown that HAS are closer in the nuclear space in males than in females, suggesting that long-range associations between MSL-complex target sites shape nuclear organization [71]. On mitotic metaphase chromosomes, the MSL-complex is only seen on the distal X-chromosome in the wild type. Surprisingly, the same pattern is seen in *roX* mutants, although the specificity of the MSL targeting is somewhat lower in these cases. We speculate that the HAS present on the X-chromosome provide superior targets for the complex when transcription is suppressed (as is the case in metaphase) compared to the centromeric regions.

Overall our results suggest that the MSL-complex has a greater affinity for *roX* RNAs than its heterochromatic targets and so *roX* RNAs restrict the targeting of the complex to the X-chromosome. In keeping with this hypothesis, a previous study showed that when MSL1 and MSL2 are overexpressed, MSL2 targets not only the X-chromosome but also some autosomal sites, the 4^th^ chromosome and the chromocenter [72]. *roX1* RNA was only detected on the X-chromosome and on some autosomal sites but not on the 4^th^ or the chromocenter. This suggests that the MSL-complex has an intrinsic affinity for heterochromatin and a balanced amount of *roX* RNAs are required to restrict the complex from these targets. An affinity for heterochromatin components may in fact be part of the mechanism to limit the activating potential of MOF when sequestered to the male X-chromosome. Although not detected in ChIP experiments or chromosome immunostainings [73]–[76], a low amount of HP1a along the entire male X-chromosome has been found in genome-wide mapping of HP1a using the DamID technique [77]. In addition, a knock-down of HP1a indicated more lethality in males than in females [78]. It is possible that when the strong binding to the X-chromosome is decreased in the *roX* mutants, the affinity for HP1a relocates the complex to canonical HP1a binding sites: heterochromatin, 4^th^ chromosome, repeat arrays.

### High Affinity Site targeting by the MSL-complex is independent of *roX* RNAs

In the absence of *roX*, the MSL-complex still targets a reduced number of sites on the X-chromosome. Based on previous cytological analysis it has been argued that these sites are similar but not identical to the sites targeted in *msl3* mutants, i.e. HAS [47], [68]. Our ChIP-seq results show an almost perfect overlap between MSL targets in *roX* mutants and the 263 previously identified HAS. In addition, the enriched sequence motif revealed by our bioinformatics analysis is nearly identical in wild type and *roX* mutants and also when comparing *D. melanogaster* and *D. simulans*. Since previous studies showed that MSL targeting to HAS is independent of MSL3, MOF and MLE, and we found HAS targeting to be independent of *roX* RNAs, we propose that MSL1 and MSL2 are the only components required for the correct targeting of HAS.

### *roX* RNAs are not required for the assembly of a complete and active MSL-complex

Interestingly, we detected a very strong MSL signal and a high enrichment of the complex in the regions surrounding six specific genes on the 4^th^ chromosome in *roX* mutants. Several lines of evidence suggest an evolutionary relationship between the 4^th^ chromosome and the X-chromosome [1], [50]–[52]. However, we cannot presently explain why these genes are specifically targeted in this way. Our findings indicate that the MSL-complexes formed at these non-X chromosome locations are complete and active, and even include associated factors such as JIL1.

Importantly we did not observe any obvious change in the expression of the six 4^th^ chromosome genes targeted by the intact MSL-complex (although this may be partly due to the limited sensitivity of qPCR). This again suggests that the activation potential of MOF within the MSL-complex is limited [17], [24], [79]. It has been shown that the targeting of MOF alone to reporter transgenes result in a strong increased expression. In contrast, when MOF was targeted as part of the MSL-complex no increased expression was observed [24]. In addition, it is important to note that the expression of all genes from the 4^th^ chromosome is fine-tuned by a balance between HP1a, which represses gene expression, and POF, the chromosome 4-specific protein that stimulates gene expression [80]–[82]. The predicted effect on gene expression due to MSL targeting to the 4^th^ chromosome in *roX* mutants might be counteracted by HP1a and/or POF. We did not observe any clear difference between *roX* mutants and the wild type with respect to the binding of POF to the 4^th^ chromosome or the binding of HP1a to either the chromocenter or the 4^th^. This demonstrates that MSL binding does not interfere with that of HP1a or POF.

### The MSL-complex has an affinity for regions enriched in *Hoppel* transposable elements and repeats

A link between dosage compensation and transposable elements has previously been suggested for both mammals and *Drosophila*. The mammalian X-chromosome is enriched in LINE elements (particularly in its pericentromeric region). It has been suggested that these elements boost the X-inactivation signal by acting as anchoring stations for the spread of the *Xist* RNA [83]. We identified a strong connection between MSL targeting and the *Hoppel* transposable element using two different approaches. The first involved identifying sequence motifs in the mapped regions, typically pericentric regions on chromosome arms and targets on the 4^th^ chromosome; this revealed a recurring sequence motif from the *Hoppel* element. The second involved calculating the enrichments of all reads, both mapped and unmapped. Strong enrichment was observed for *PROTOP_A* and *PROTOP_B* (both corresponding to *Hoppel* [59]) as well as *NTS Dm*. Notably, *NTS* is enriched by MOF also in wild type suggesting that this is an intrinsic target of MOF which is stabilized by the complete MSL-complex in *roX* mutants. The importance of *Hoppel* in MSL targeting is supported by its high degree of colocalization with MSL staining in *roX* mutant males. Because the ChIP technique relies on the analysis of fractionated DNA, we cannot currently say whether it is *Hoppel* itself or transcribed regions in its vicinity that are targeted by MSL. The *Hoppel* elements are non-autonomous DNA transposons and among the most abundant transposable elements in the *D. melanogaster* genome, being enriched in the pericentric heterochromatin and on the fourth chromosome [84]. Since we also observed MSL enrichment in *NTS* (Non-Transcribed Spacer) regions, active autosomal genes surrounded by DNA repeats, and tandemly repeated gene constructs (*P[lacW]*), it seems that MSL is recruited to repeats in general. A recent study demonstrated that the neo-X of *D. miranda* has newly evolved chromatin entry sites (CES), also known as high affinity sites-HAS, that recruit MSL and are enriched in the ISX helitron transposable element (TE) [85]. The authors suggest that the evolutionary acquisition of the MSL-complex by X-chromosomes involved the acquisition of GA-rich sequence motifs by transposable elements that were capable of functioning as HAS for the MSL-complex, followed by amplification of the TEs across the genome. This may then have been followed by positive selection for these elements on the X-chromosome followed by a refinement process that eroded TEs in non-functional regions and increased their affinity for MSL. The authors further suggest that the heterochromatization of the neo-Y occurs in parallel with the acquisition of dosage compensation on the neo-X.

We speculate that the MSL targeting seen in the absence of *roX* RNAs represents an ancient but still intrinsic property of the MSL-complex. This model suggests that *roX* RNAs are younger in evolutionary terms than the protein components of the MSL-complex and evolved in parallel with the degeneration of the Y-chromosome, redistributing the MSL-complex to the male X-chromosome and restricting its intrinsic heterochromatic targeting. In fact, a human MSL-complex (hMSL) has been identified that contains the homologs of the *Drosophila* proteins MSL1, MSL2, MSL3 and MOF, indicating that the MSL-complex protein components are highly conserved in evolution. Conversely, the *roX* RNAs evolve rapidly [30]. The hMOF is responsible for the majority of H4K16 acetylation as well as being involved in the acetylation of other substrates such as the p53 protein, and in the regulation of various cellular processes (reviewed in [86], [87]). The ancient function of MSL in the heterochromatin of a *Drosophila melanogaster* ancestor may have been to activate the expression of active genes present in repressive environments. The binding of MSL to *NTS* in *roX* mutants supports the hypothesis that MSL may have had a role in protecting active genes that are present in multiple copies in the genome, like the ribosomal genes, against repeat-induced gene silencing [83], [88]. This is also supported by our finding that active genes on autosomes bound by MSL in *roX* mutants are in repeat-enriched regions. It has been shown that sequences containing *P[lacW]* in tandem repeats become heterochromatic and the repeated mini-*white* gene becomes partially repressed [64]. MSL is recruited to this transgene in a *roX* mutant background suggesting that it is recruited to repeat-induced gene silencing regions.

## Materials and Methods

### Fly stocks and genetic crosses

Flies were cultivated and crossed in vials containing potato mash-yeast-agar medium at 25 C. The wild type strains used were *D. melanogaster (Oregon R)* and *D. simulans/w^501^* (UC San Diego *Drosophila* Stock Center). The *D. melanogaster roX1 roX2* double-mutant males were selected as non-GFP males from a *y w roX1^ex6^ Df(1)roX2^52^ P[w^+^ 4Δ4.3]/FM7i, P[w^+mC^ ActGFP]JMR3* stock obtained from Yongkyu Park (New Jersey Medical School, Newark, NJ). The *mof* mutants were obtained by crossing virgin females from the stock *mof^2^; P[w^+^ mof^+^]/CyO GFP*, obtained from Peter Becker (Ludwig Maximilians Universität Munchen), to wild type males and selecting green-fluorescent males in the progeny. The *mle* mutants were obtained by selecting the non-green fluorescent males from the cross: *mle^9^ cn^1^ bw^1/^CyO, P[w^+mC^ ActGFP]JMR1* ×*FM7i, P[w^+mC^ = ActGFP]JMR3/Y; mle^1^/CyO, P[w^+mC^ ActGFP]JMR1*. The strains carrying the *P[lacW]* transgene in repeats *C-2, BX-2, T-1,* and *1A-6* were kindly provided by Stephane Ronsseray (CNRS-Université Pierre et Marie Curie) and are described elsewhere [64], [89], [90]. Insertions on the second chromosome were rebalanced with *CyO, P[w^+mC^ ActGFP]JMR1*, the rebalanced males were crossed to *y w roX1^ex6^ Df(1)roX2^52^ P[w^+^ 4Δ4.3]/FM7i, P[w^+mC^ ActGFP]JMR3* females, and salivary glands were dissected from non-GFP male larvae. To study the targeting of MSL to *lac* repeats we used the strains *P[hs-HP1.lacI.BD]* and *P[Ecol\lacO.256x.w]157.4.112* [65], kindly provided by Lori Wallrath (University of Iowa). *roX1^ex6^ Df(1)roX2^52^ P[w^+^4Δ4.3]/Y;P[Ecol\lacO.256x.w]157.4.112/+; P[hs-HP1.lacI.BD]/+* males were obtained by crossing *w; P[Ecol\lacO.256x.w]157.4.112* males with *roX1^ex6^ Df(1)roX2^52^ P[w+4Δ4.3]/FM7i; P[hs-HP1.lacI.BD]* females. Third instar larvae were heat-shocked for 45 minute at 37 C and recovered at room temperature for 2–3 h prior to dissection.

### Transgenic flies

To generate transgenic flies carrying a transgene with repeats of the *ankyrin* gene together with the motif found to be enriched at heterochromatic sites bound by MSL in *roX* mutants, a DNA fragment containing the *attB* integration site was excised from *pTA-attB* [91] with *Eco*RI and cloned into the *CaSpeR-4* vector. The resulting *pCas-attB* plasmid was used as a cassette for integrating *ankyrin* cDNA downstream of a 108 nucleotide-long DNA fragment identical to the *Hoppel* element 1360{}6073 (FBti0064134), repeated three times. A plasmid containing this *Hoppel* repeat was produced by GenScript USA Inc. The repeat was excised with *Kpn*I and cloned into *pCas-attB* (*pCas-attB-1360*). A cDNA clone of *ank-RB* (*LD10053*) was purchased from the *Drosophila* Genomic Research Center. The desired DNA fragment was excised with *Not*I and *Xho*I and cloned into *pCas-attB-1360* digested with the same nucleases. Finally, the promoter region of *ank* was amplified with the primers 5′-atagcggccgcttaggtatgtaaaattcacgcaa-3′ and 5′-cgagcggccgcaaggcaggctcaggtatttg-3′, digested with *Not*I and cloned upstream the *ank-RB* fragment. Embryo microinjection into the *Bl9750* strain (3L:65B2 PhiC landing platform) was performed by BestGene (Inc). Males homozygous for the transgene were crossed to *y w roX1^ex6^ Df(1)roX2^52^ P[w^+^ 4Δ4.3]/FM7i, P[w^+mC^ ActGFP]JMR3* females and salivary glands were dissected from non-GFP male larvae.

### Immunostainings and DNA *in situ* hybridization (DNA-FISH)

Third instar larvae polytene chromosomes from salivary glands were prepared as described previously [92]. Larval brain squashes were performed according to protocol 1.9, method 3 in [93]. Immunostainings were performed as described previously [94] with the following antibodies (dilutions in parentheses): rabbit anti-MSL1 (1∶400), MSL2 (1∶200), MOF (1∶400) and MLE (1∶2000), and goat anti-MSL3 (1∶2000) from Mitzi Kuroda (Harvard Medical School); rabbit anti-JIL1 (1∶1000) from Peter Becker (Ludwig Maximilians Universität Munchen); and rabbit anti-H4K16ac (1∶300, sc-8662-R, Santa Cruz). The secondary antibodies used were donkey anti-goat or donkey anti-rabbit conjugated with AlexaFluor555 or AlexaFluor488, respectively (1300 dilution, Molecular Probes) together with DAPI (1 µg/ml). DNA-FISH combined with immunostaining on polytene chromosomes and brain squashes was performed according to a standard protocol [95]. The probe against mini-*white* was excised from *CaSpeR-4* plasmid using the *EcoRI* restriction endonuclease and biotin labelled with the BioNick DNA Labeling System (Life Technologies). A FAM-labelled probe against 1.686 g/cm^3^ satellite was purchased from Exiqon. The sequence of the 33 nucleotide-long biotin-labelled probe that was used against the heterochromatic motif found to be enriched at MSL-bound regions in *roX* mutants was 5′-TAACAAGATGCGTAACGGCCATACATTGGTTTG-3′. Antibodies for the detection of DNA probes were mouse anti-FITC and mouse anti-biotin (1∶500, Jackson ImmunoResearch) with goat anti-mouse labelled with AlexaFluor488 as secondary antibody. HP1a was detected with rabbit PRB291C antibody (1∶400, Covance) and with donkey anti-rabbit AlexaFluor555. Preparations were analyzed using a Zeiss Axiophot microscope equipped with a KAPPA DX20C CCD camera. For comparisons between strains or proteins stained, the protocol was run in parallel. Nuclei with clear cytology were chosen on the basis of DAPI staining and photographed. At least 20 nuclei for each genotype were used in these comparisons, and at least four slides of each genotype were analyzed. For the colocalization analysis of the 1686 probe DNA/FISH combined with MSL3 immunostaining, 8 biological replicates (8 slides with one brain per slide) from each of the wild type, *mof* mutants, and *roX1 roX2* mutants were analyzed with 30–50 nuclei scored per replicate. Nuclei were chosen on the basis of DAPI staining and colocalization was scored.

### Quantitative Real-time PCR

Total RNA was extracted from third instar larvae using TRI reagent (Ambion) according to the manufacturer's protocol. Three biological replicates from the wild type and *roX1 roX2* mutants were produced, consisting of 10 male larvae each. The RNA was reverse-transcribed using the iScript cDNA Synthesis kit (Bio-Rad) and amplified by real-time PCR using iQSYBR Green Supermix (Bio-Rad) according to the manufacturer's instructions. Primer pairs used are listed in Supplementary Table S1. The expression levels were normalized to the amount of *RpL32* mRNA in each replicate.

### Chromatin immunoprecipitation and deep sequencing (ChIP-seq)

The ChIP experiments were performed in salivary glands from third instar larvae as previously described [80], [81] using 3 µl of anti-MSL1, 3 µl of anti-MOF and 2 µl of anti-MSL2 (provided by Mitzi Kuroda, Harvard Medical School). To verify the quality of the input and ChIP samples before sequencing, we analyzed the ChIP DNA/input DNA ratio, using real time PCR as described previously [81]. We generated one replicate of MSL1, MOF and MSL2 for each genotype (*D. simulans* wild type, *D. melanogaster* wild type and *D. melanogaster roX1 roX2* homozygous mutant). Library preparation and AB SOLiD 5500xl sequencing were performed by Uppsala Genome Centre. The MSL1 sample from the *D. melanogaster* wild type was unfortunately lost. The complete dataset is available at http://www.ncbi.nlm.nih.gov/geo/ (Accession: GSE58768).

### ChIP-seq data processing

Uniquely mapped reads from all samples were aligned against the *D. melanogaster* (*Dm*) reference sequence (release 5) and *D. simulans (Ds)* reference sequence (release 1) using the Applied Biosystems Bioscope software v1.2.1. Enrichment ratios for the MSL1, MSL2 and MOF proteins in *Dm* and *Ds* wild type and *Dm roX1 roX2* mutant samples were calculated as described previously [96]. Ratio values every 10 bp were extracted across the genome and median smoothed using a window size of 500 bp or 2000 bp; windows with fewer than 25 and 100 data points, respectively, were discarded. Since the MSL1 enrichment ratios for the *roX1 roX2* mutant samples and the MSL2 enrichment ratios for the wild type samples were the most distinct, these samples were selected as the representative ones in the wild type and *roX1 roX2* mutant groups. To define the MSL-bound regions, the highest 1.5 percent of the ratio values were extracted. Data units that crossed this cutoff and that are spaced no more than 200 bp from each other were then combined into MSL-bound regions. Regions of less than 200 bp or containing fewer than five data units were discarded. Each bound region was assigned a value equal to the average of the top five consecutive ratio values. The MSL peak centre for each MSL bound region was set to the centre position of the top five consecutive ratio values.

### Distance analysis

The 263 high affinity sites (HAS) as defined in [34], [37], [38] were used to calculate the closest distance to the MSL1-bound regions (defined as above) on the X-chromosome in *roX* mutants. The distances were divided into 8 bins and the fraction of MSL1-bound sites in each bin was calculated. As a control we used the distance between the HAS and random locations on the X-chromosome.

### Motif analysis

In order to search for HAS motif in our ChIP-seq data, 200 bp regions around the centres of peak MSL1 abundance on the X-chromosome in *roX* mutants were analysed with the MEME program [97] using default parameters. Similar analyses were also performed for the top 200 MSL1-bound regions on the X-chromosome in the *Ds* wild type.

In order to search for DNA motifs enriched in heterochromatic regions bound by MSL in *roX* mutants, 200 bp regions around the centres of peak MSL1 abundance on the heterochromatin scaffolds of each chromosome (2LHet, 2RHet, 3LHet, 3RHet, 4Het, XHet) in *roX* mutants were analysed with the MEME program [97] together with scrambled sequences of binding sites as negative sequences, using default parameters.

### Repeat analysis

In order to analyse the repeat content in regions surrounding the expressed genes (defined in [76]) overlapping with MSL1 bound/unbound regions, repeat masked sequences were downloaded from UCSC [98],[99] and the fractions of repeat masked nucleotides in 200 bp windows at 10 bp intervals across the genome were calculated. The percentages of these repeats in 20 bins of 1 kb around the Transcription Start Site (TSS) of MSL1 bound/unbound genes in *roX* mutants, on autosomes and on the X-chromosome were then calculated. Repeat percentages were also calculated around the TSS of all expressed genes of the X-chromosome and autosomes and around MSL2 bound/unbound expressed genes in the wild type, filtered using a 5 percent highest ratio cutoff on MSL2 enrichment ratio values.

To search for repeat classes enriched in MSL-bound ChIP-seq reads, repeat classes in *Dm* available from the Repbase Update database (release 19.01) [100] were used. Reads from wild type and *roX* mutant as well as the corresponding inputs were mapped to different repeat classes using the Bowtie software parameters –a (to map all reads) –v 2 (with two mismatches) [101]. For each repeat class, an RPKM value (*R*eads *P*er *K*ilobase per *M*illion mapped reads) [102] was calculated which was used further to calculate a ratio between ChIP/input in wild type and *roX* mutants, respectively. The number of reads that mapped to the genome in the original ChIP-seq analysis was used as the number of mapped reads. In each repeat class, read counts per nucleotide was also calculated from wild type and *roX* mutant as well as input, and normalized to the number of mapped reads (in millions) from each sample.

## Supporting Information

S1 Figure H4K16ac and MSL3 immunostaining on polytene chromosomes from *roX* mutant males, showing the 4^th^ chromosome and chromocenter targeting. Note that H4K16ac on the 4^th^ chromosome shows a broader enrichment pattern compared to the MSL proteins in similarity to what previously have been observed for H4K16ac in relation to MSL on the male X-chromosome in wild type.
(PDF)

S2 Figure Female/male ratio of reads coverage from DNA-seq [50]. (A) Average for all genes of each chromosome. (B) Average for the six genes targeted by MSL in *roX* mutants, and for the remaining 4^th^ chromosome genes.
(PDF)

S3 Figure DNA-FISH with a probe against the mini-*white* gene (marker on *pCas-attB-1360*) combined with MSL2 immunostaining, on polytene chromosomes from *roX* mutant males carrying a transgene with the *ankyrin* cDNA downstream of three tandem repeats of 1360{}6073, identical to the *Hoppel* element.
(PDF)

S4 Figure MSL3 and HP1a immunostaining on polytene chromosomes from roX mutant males carrying a transgene with 256 tandem repeats of *lacO* gene and another transgene coding for the protein fusion HP1-lacI.BD, which is tethered to *lacO* (*roX1^ex6^ Df(1)roX2^52^ P[w^+^4Δ4.3]/Y;P[Ecol\lacO.256x.w]157.4.112/+; P[hs-HP1.lacI.BD]/+*).
(PDF)

S1 Table List of primer pair sequences used in the quantitative real-time PCR experiment.
(PDF)
