# Supplementary figures and images for: Non-coding roX RNAs Prevent the Binding of the MSL-complex to Heterochromatic Regions
Source: PLoS Genet. 2014 Dec 11;10(12):e1004865. doi: 10.1371/journal.pgen.1004865 (PMC4263465; doi:10.1371/journal.pgen.1004865)

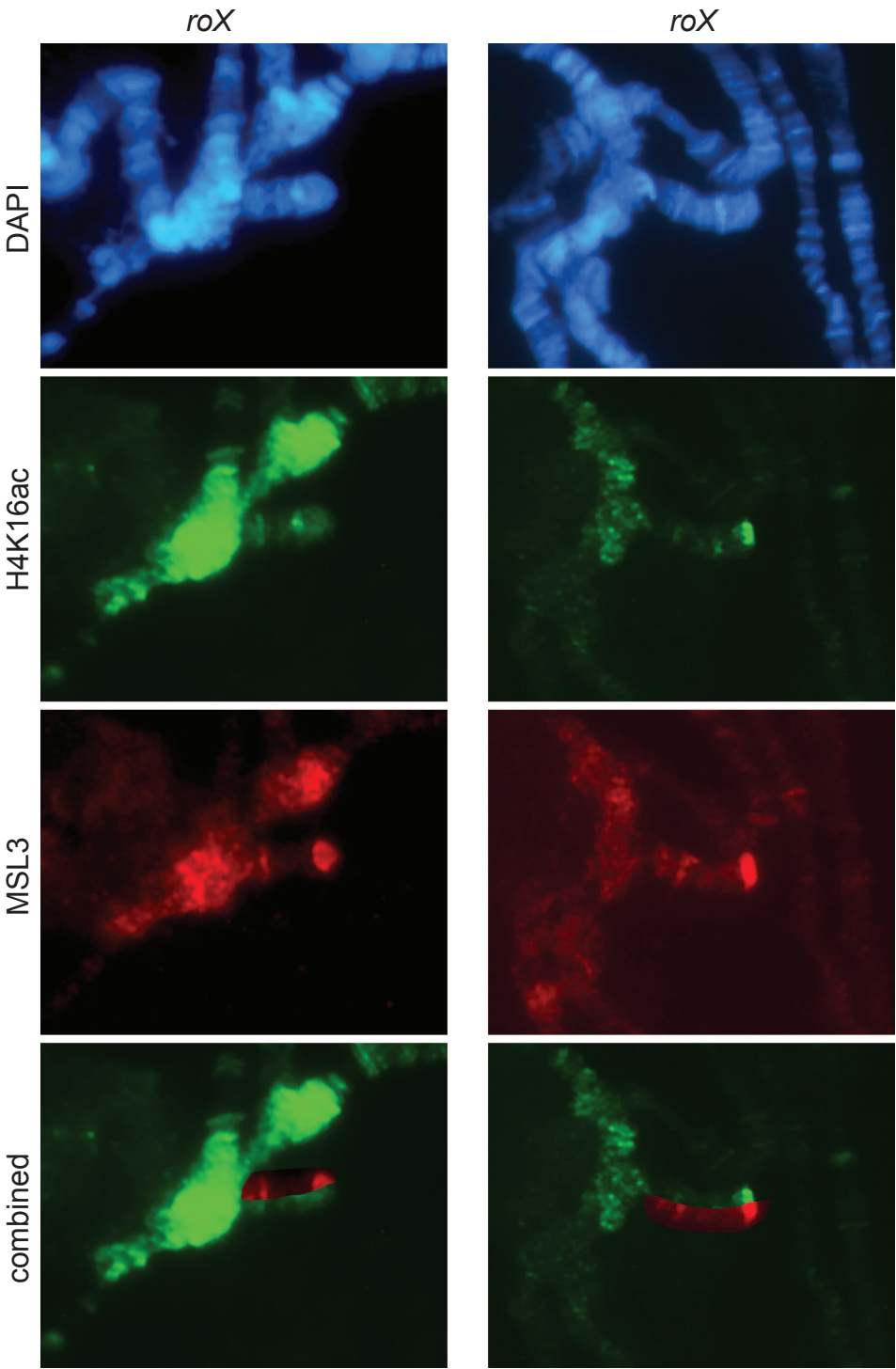

Supplement: S1 Figure — H4K16ac and MSL3 immunostaining on polytene chromosomes from roX mutant males, showing the 4th chromosome and chromocenter targeting. Note that H4K16ac on the 4th chromosome shows a broader enrichment pattern compared to the MSL proteins in similarity to what previously have been observed for H4K16ac in relation to MSL on the male X-chromosome in wild type. (PDF) [file pgen.1004865.s001.pdf]

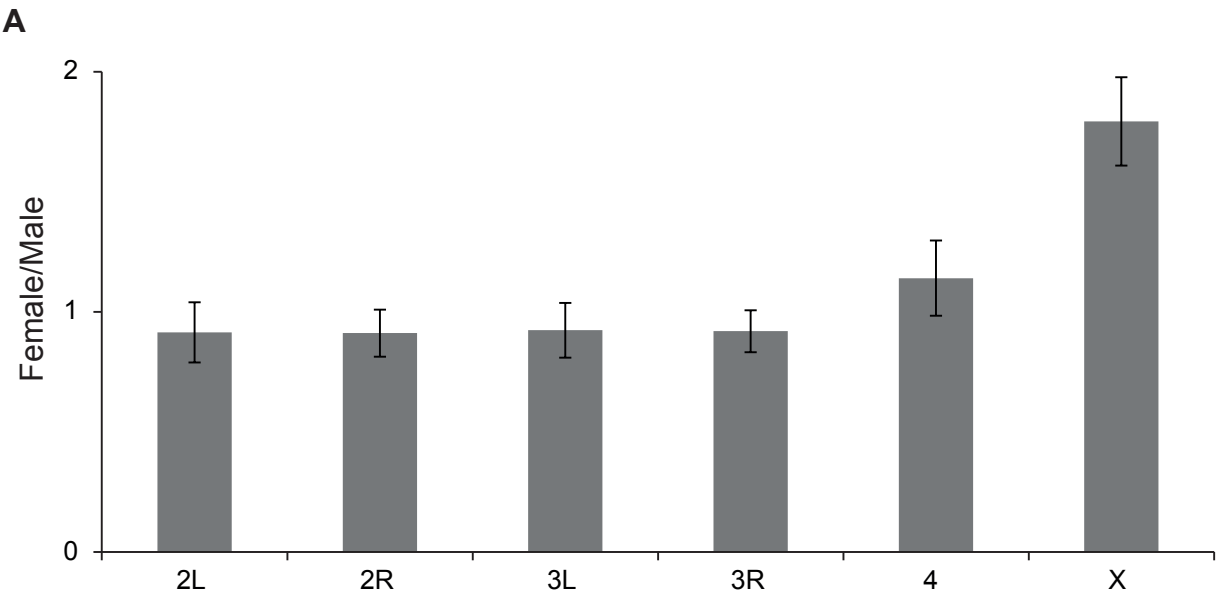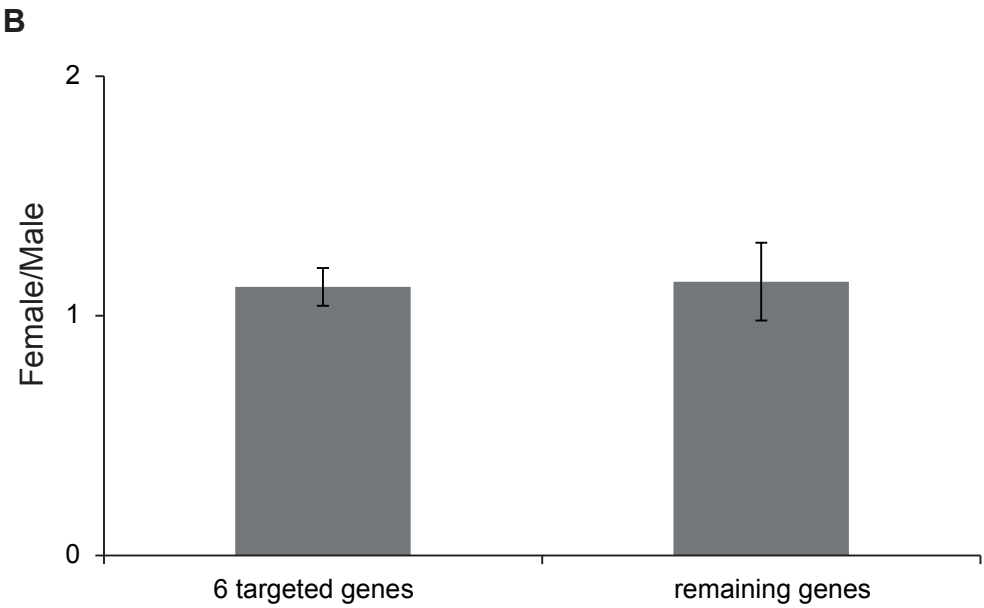

Supplement: S2 Figure — Female/male ratio of reads coverage from DNA-seq [50]. (A) Average for all genes of each chromosome. (B) Average for the six genes targeted by MSL in roX mutants, and for the remaining 4th chromosome genes. (PDF) [file pgen.1004865.s002.pdf]

DAPI

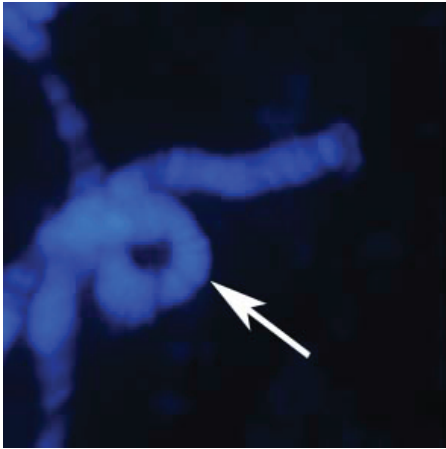

FISH

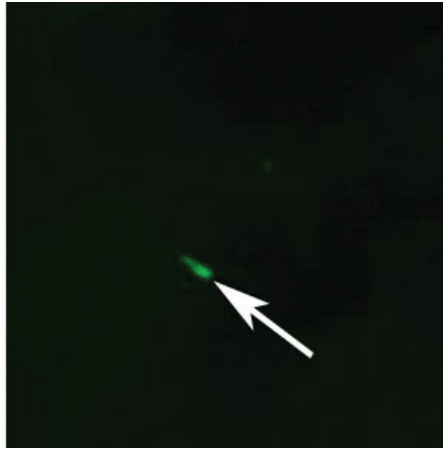

MSL2

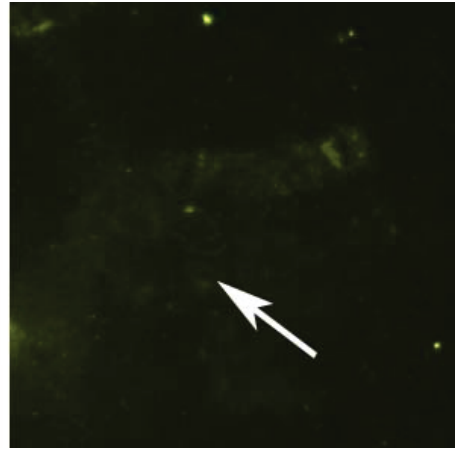

Supplement: S3 Figure — DNA-FISH with a probe against the mini-white gene (marker on pCas-attB-1360) combined with MSL2 immunostaining, on polytene chromosomes from roX mutant males carrying a transgene with the ankyrin cDNA downstream of three tandem repeats of 1360{}6073, identical to the Hoppel element. (PDF) [file pgen.1004865.s003.pdf]

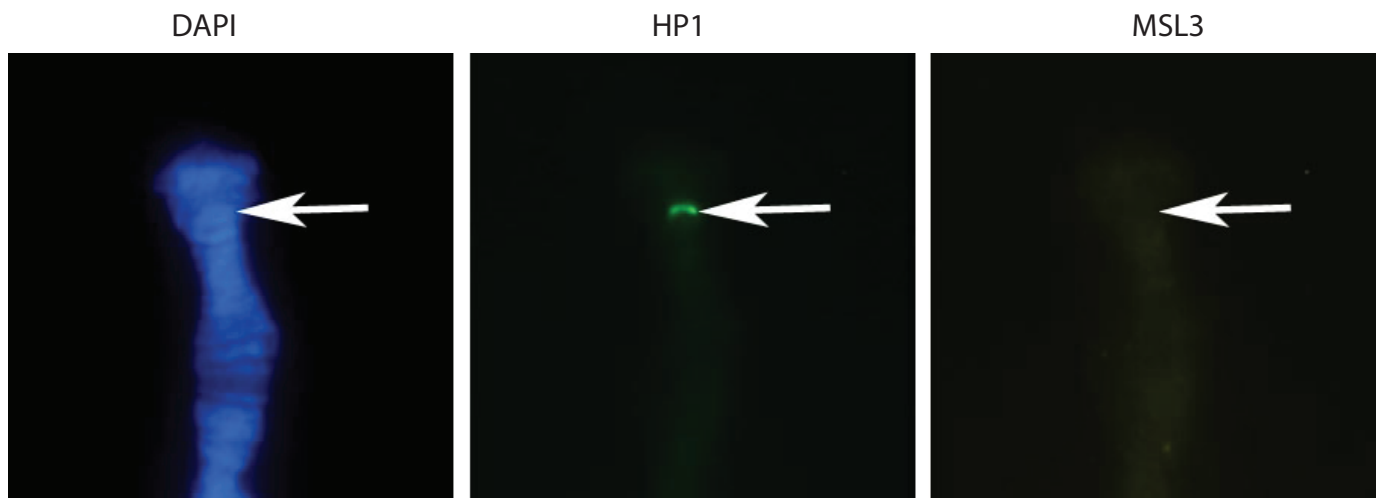

Supplement: S4 Figure — MSL3 and HP1a immunostaining on polytene chromosomes from roX mutant males carrying a transgene with 256 tandem repeats of lacO gene and another transgene coding for the protein fusion HP1-lacI.BD, which is tethered to lacO (roX1ex6 Df(1)roX252 P[w+4Δ4.3]/Y;P[Ecol\lacO.256x.w]157.4.112/+; P[hs-HP1.lacI.BD]/+). (PDF) [file pgen.1004865.s004.pdf]
