# Supplementary material for: Non-coding roX RNAs Prevent the Binding of the MSL-complex to Heterochromatic Regions
Source: PLoS Genet. 2014 Dec 11;10(12):e1004865. doi: 10.1371/journal.pgen.1004865 (PMC4263465; doi:10.1371/journal.pgen.1004865)
Supplement: S1 Table — List of primer pair sequences used in the quantitative real-time PCR experiment. (PDF) [file pgen.1004865.s005.pdf]

Supplementary Table S1

	Sense	Antisense
Ankyrin	5’-GGTGTTTCTCCGCGTATTG-3’	5’-AGCTAACATTGGCGCAAC-3’
Rad23	5’-CAATAGGCTGAATGAATCAACG-3’	5’-TTTGAAGAAACTGTGGCTGG-3’
CG2177	5’-ACGTCTTGATGGCTAGTGG-3’	5’-TTGGTGGACATCTCTCAGC-3’
PMCA	5’-CCATTGGTTCAATAACTTCACG-3’	5’-CGTCCTAACGGAGGATATCG-3’
Dyrk3	5’-ATACGCTGGTTCTCGAAGC-3’	5’-CTTTGCTACAATGTTTGGATGC -3’
Mitf	5’-GCTACCATCATTCGACAGC-3’	5’-TTGGCTAGGGCATTCATTTC-3’
MED26	5’-TCGATGTAGCATCTCCAGATAC-3’	5’-AAAGAGAAGGACAGGTCGC-3’
RpL32	5’-CGATGTTGGGCATCAGATAC-3’	5’-CCCAAGATCGTGAAGAAGC-3’
